# Generation, Transcriptomic States, and Clinical Relevance of CX3CR1^+^ CD8 T Cells in Melanoma

**DOI:** 10.1158/2767-9764.CRC-24-0199

**Published:** 2024-07-24

**Authors:** Hirohito Ishigaki, Takayoshi Yamauchi, Mark D. Long, Toshifumi Hoki, Yuta Yamamoto, Takaaki Oba, Fumito Ito

**Affiliations:** 1 Department of Surgery, University of Southern California, Norris Comprehensive Cancer Center, Los Angeles, California.; 2 Division of Pathogenesis and Disease Regulation, Department of Pathology, Shiga University of Medical Science, Otsu, Japan.; 3 Center for Immunotherapy, Roswell Park Comprehensive Cancer Center, Buffalo, New York.; 4 Department of Biostatistics & Bioinformatics, Roswell Park Comprehensive Cancer Center, Buffalo, New York.; 5 Oncology Science Unit, MSD Japan, Tokyo, Japan.; 6 Department of Surgery, Shinshu University School of Medicine, Matsumoto, Japan.

## Abstract

Recent progress in single-cell profiling technologies has revealed significant phenotypic and transcriptional heterogeneity in tumor-infiltrating CD8^+^ T cells. However, the transition between the different states of intratumoral antigen-specific CD8^+^ T cells remains elusive. Here, we sought to examine the generation, transcriptomic states, and the clinical relevance of melanoma-infiltrating CD8^+^ T cells expressing a chemokine receptor and T-cell differentiation marker, CX3C chemokine receptor 1 (CX3CR1). Analysis of single-cell datasets revealed distinct human melanoma-infiltrating CD8^+^ T-cell clusters expressing genes associated with effector T-cell function but with distinguishing expression of* CX3CR1 or PDCD1*. No obvious impact of *CX3CR1* expression in melanoma on the response to immune checkpoint inhibitor therapy was observed while increased pretreatment and on-treatment frequency of a CD8^+^ T-cell cluster expressing high levels of exhaustion markers was associated with poor response to the treatment. Adoptively transferred antigen-specific CX3CR1^−^ CD8^+^ T cells differentiated into the CX3CR1^+^ subset in mice treated with FTY720, which inhibits lymphocyte egress from secondary lymphoid tissues, suggesting the intratumoral generation of CX3CR1^+^ CD8^+^ T cells rather than their trafficking from secondary lymphoid organs. Furthermore, analysis of adoptively transferred antigen-specific CD8^+^ T cells, in which the *Cx3cr1* gene was replaced with a marker gene confirmed that CX3CR1^+^ CD8^+^ T cells could directly differentiate from the intratumoral CX3CR1^−^ subset. These findings highlight that tumor antigen–specific CX3CR1^−^ CD8^+^ T cells can fully differentiate outside the secondary lymphoid organs and generate CX3CR1^+^ CD8^+^ T cells in the tumor microenvironment, which are distinct from CD8^+^ T cells that express markers of exhaustion.

**Significance:**

Intratumoral T cells are composed of heterogeneous subpopulations with various phenotypic and transcriptional states. This study illustrates the intratumoral generation of antigen-specific CX3CR1^+^ CD8^+^ T cells that exhibit distinct transcriptomic signatures and clinical relevance from CD8^+^ T cells expressing markers of exhaustion.

## Introduction

CD8^+^ T cells play an important role in immune defense and the control of viral infections and cancer (1). Adoptive T-cell therapy, involving *ex vivo* expansion and infusion of T cells containing tumor-specific CD8^+^ T cells, such as chimeric antigen receptor T (CAR-T) cells and tumor-infiltrating lymphocytes (TIL), has become a potent treatment modality for advanced hematological and solid malignancies refractory to conventional therapy (2, 3). The *in vivo* expansion and persistence of adoptively transferred CD8^+^ T cells are important determinants of antitumor responses (4–7), whereas the immunosuppressive tumor microenvironment limits the trafficking and effector function of infused T cells (8–10). Compelling evidence has shown that less-differentiated antigen-specific T cells persist longer, traffic more effectively to the tumor microenvironment, and mediate greater antitumor reactivity compared with fully differentiated effector T cells (4–7). Consistent with this, memory- or stem-like T cells expressing T-cell factor-1 (TCF-1), CD62L, CD27, and/or CD127 were present in tumors responding not only to adoptive T-cell therapy but also to immune checkpoint inhibitor (ICI) therapy (11). Fully differentiated CD8^+^ T cells can be identified in the tumor microenvironment; however, their clinical relevance remains unclear.

CX3C chemokine receptor 1 (CX3CR1) is a receptor of fractalkine (CX3CL1) for trafficking and adhesion to inflammatory sites and is expressed on the surface of immune cells, such as T cells, natural killer (NK) cells, monocytes, tissue-resident dendritic cells (DC), and macrophages (12, 13). CX3CR1 was also found to be a marker of T-cell differentiation, where functionally distinct viral and tumor-specific CX3CR1^−^ CD8^+^ T cells expressing high levels of TCF-1, CD62L, CD27, and/or CD127 can give rise to CX3CR1^+^ subsets via unidirectional differentiation upon activation *in vivo* (14, 15). Consequently, CX3CR1^+^ CD8^+^ T cells increase in the periphery after effective immunotherapies, and T-cell CX3CR1 expression acts as a predictive marker of response in preclinical models and patients (15–22). CX3CR1^+^ T-cell subsets can be identified in tumors (15, 17); however, it remains unclear whether they migrate from secondary lymphoid organs via the CX3CR1/CX3CL1 axis or directly differentiate from CX3CR1^−^ CD8^+^ T cells in the tumor microenvironment. Additionally, their transcriptomic signatures in relation to “exhausted” CD8^+^ T cells remain elusive.

In this study, we evaluated the frequency, transcriptomic states, and clinical relevance of CX3CR1^+^ CD8^+^ T cells in human melanoma using resected specimens and publicly available single-cell data. Furthermore, using a preclinical model of melanoma, we sought to determine whether tumor antigen-specific CD8^+^ T cells could differentiate within tumors or traffic from secondary lymphoid organs. To this end, we used FTY720, which inhibits the emigration of T cells from secondary lymphoid organs, to examine T-cell differentiation outside the lymphoid tissues. Furthermore, we generated a new mouse model in which the gene *Cx3cr1* was homozygously deleted and replaced with the diphtheria toxin receptor (DTR) in antigen-specific CD8^+^ T cells (Pmel-1 *Cd2-cre/Cx3cr1*^*DTR/DTR*^ mice) to definitively assess the ability of intratumoral differentiation of tumor-specific CD8^+^ T cells *in vivo*. Our data revealed that tumor antigen–specific CX3CR1^−^ CD8^+^ T cells can fully differentiate outside the secondary lymphoid organs and generate CX3CR1^+^ CD8^+^ T cells in the tumor microenvironment.

## Materials and Methods

### Ethics statement

All animal studies were approved by the Roswell Park Institutional Animal Care and Use Program and Facilities (protocols 1316M and 1356M) and conducted in accordance with the Federal Animal Welfare Act and NIH’s Guide for the Care and Use of Laboratory Animals (National Academies Press, 2011). De-identified human melanoma samples were obtained from patients with written informed consent under the protocol (BDR 111519) approved by the Institutional Review Board of the Roswell Park Comprehensive Cancer Center.

### Human tumor sample dissociation

Freshly resected tumors were collected immediately after surgery and processed within 1 hour as previously described (15). Briefly, tumors were dissected away from adjacent normal tissue and stroma, incubated with a tumor dissociation kit (Miltenyi Biotec) in C Tubes (Miltenyi Biotec), and dissociated using a GentleMACS dissociator (Miltenyi Biotec). Tumor and infiltrating cells were filtered through a cell strainer (70 μm; BD Biosciences) and single-cell suspensions were obtained.

### Mice

C57BL/6 mice, CD2-Cre mice [C57BL/6-Tg(CD2-cre)1Lov/J], Pmel-1 T-cell receptor (TCR)-transgenic mice [B6.Cg *Thy1*^*a*^-Tg(TcraTcrb)8Rest/J)], and CX3CR1-DTR mice (B6N.129P2-Cx3cr1^*tm3(DTR)Litt*^/J) were purchased from The Jackson Laboratory and/or bred in-house (Roswell Park Comprehensive Cancer Center). Generation of inducible Pmel-1 *Cd2-cre/Cx3cr1*^*DTR/DTR*^ mice: Pmel-1 mice were crossed with CD2-Cre mice (Supplementary Fig. S1A). Next, Pmel-1^+/−^*Cd2-cre* mice were backcrossed with Pmel-1 mice to generate Pmel-1^+/+^*Cd2-cre* mice. Pmel-1^+/+^*Cd2-cre* mice were crossed with CX3CR1-DTR mice to generate Pmel-1 *Cd2-cre/Cx3cr1*^*+/DTR*^ mice (Supplementary Fig. S1B) as described previously (15). Finally, we crossed Pmel-1 *Cd2-cre/Cx3cr1*^*+/DTR*^ mice with Pmel-1 *Cd2-cre/Cx3cr1*^*+/DTR*^ mice to generate Pmel-1 *Cd2-cre/Cx3cr1*^*DTR/DTR*^ mice (Supplementary Fig. S1C). All mice used in this study were female on a C57BL/6 background, 7 to 10 weeks old at the beginning of each experiment, and were maintained under specific pathogen-free conditions at the Roswell Park animal facility, according to approved institutional guidelines.

### Cell lines

The murine B16F10 melanoma cell line was purchased from the ATCC. B16F10 cells were cultured in RPMI (Gibco, Thermo Fisher Scientific) containing 10% FBS (MilliporeSigma), 1% nonessential amino acids, 2 mmol/L GlutaMAX-1, 100 U/mL penicillin-streptomycin, and 55 µmol/L 2-mercaptoethanol (Gibco, Thermo Fisher Scientific). Cells were maintained in a humidified atmosphere containing 5% CO_2_ at 37°C.

### Treatment regimens (adoptive cell therapy, vaccination, cytokine administration)

Adoptive T-cell therapy for established tumors was performed as previously described (15, 23, 24). In brief, 5 × 10^5^ B16F10 cells were subcutaneously (s.c.) injected into the left flank of the female mice. Mice received 500 cGy of systemic irradiation 1 day before T-cell infusion. After culturing with murine (m) IL7 (10 ng/mL; PeproTech) and mIL-15 (10 ng/mL; PeproTech) in the presence of 1 µmol/L human (h)gp100_25–33_ peptide, KVPRNQDWL (GenScript) for 6 days, 1 × 10^6^ to 3 × 10^6^ activated Pmel-1 or Pmel-1 *Cd2-cre/Cx3cr1*^*DTR/DTR*^ splenocytes were intravenously (i.v.) injected into the tumor-bearing mice 12 to 14 days after tumor inoculation. The mice were administered 20,000 to 40,000 IU recombinant human IL2 (rhIL2; Prometheus Laboratories Inc.) intraperitoneally (i.p.) once on the day of T-cell injection and twice a day for the following 2 days. For *in vivo* activation, 100 μL of phosphate-buffered saline containing 100 μg of hgp100 peptide, 50 μg of CD40-specific antibody (clone FGK4.5, Bio X Cell), and 50 mg of imiquimod cream 5% (Perrigo) were also administered.

### Treatment with FTY720

FTY720 (Sigma) was administered to the mice to inhibit lymphocyte emigration from the secondary lymphoid organs as described previously (19, 20). Briefly, mice received a daily dose of 20 μg FTY720 diluted in 3% Tween-20 i.p. or vehicle (3% Tween-20) for 10 days, starting 6 hours before adoptive cell therapy.

### Flow cytometry assays

The fluorochrome-conjugated antibodies used for flow cytometry assays are shown in Supplementary Table S1. DTR-expressing Pmel-1 *Cd2-cre/Cx3cr1*^DTR/DTR^ T cells were stained with an anti-DTR (human HB-EGF) antibody (R&D Systems), followed by Alexa Fluor 647 AffiniPure F(ab')₂ Fragment Donkey Anti-Goat IgG (H+L; Jackson ImmunoResearch). Cells stained with DAPI or LIVE/DEAD Fixable Aqua (Thermo Fisher Scientific) were excluded from analysis. Intracellular granzyme A (GZMA), granzyme B (GZMB), IFNγ, and TNFα assays of tumor-infiltrating immune cells incubated with hgp100 peptide and GolgiStop (BD Biosciences) for 4 to 6 hours were performed using the Fixation/Permeabilization Solution Kit (BD Biosciences) according to the manufacturer’s recommendations. The samples were analyzed using LSRII or LSRFortessa (BD Biosciences) with FlowJo software (Tree Star). The gating strategies are shown in Supplementary Figs. S2 and S3.

### Analysis of melanoma single-cell RNA-sequencing data

Processed normalized single-cell RNA-sequencing (scRNA-seq) counts (log_2+1_ TPM) derived from cells captured from 48 tumor samples from patients with melanoma treated with ICIs (11) were obtained directly from the gene expression omnibus (GSE120575). Filtering and downstream analyses including variable feature selection, dimensionality reduction (principal component analysis), uniform manifold approximation and projection (UMAP) low-dimensional representation, and k-nearest neighbors (kNN)-based clustering were performed using Seurat (v4; ref. 25). Cells were mapped to the Azimuth human PBMC reference (26) as we described recently (27) and final curated cluster annotations to major immune cell lineages made in conjunction with typical linage marker assessment (Supplementary Fig. S4). T and NK cells were filtered from total cells and reanalyzed as described above for assessment of CX3CR1 in relation to various markers of T-cell phenotype. Differential expression of select T-cell markers between CX3CR1 positive and negative cells was determined by Wilcoxon rank sum test.

### Data availability

Publicly available processed single-cell data generated by Sade-Feldman et al. (11) used in this paper are available in the gene expression omnibus under the accession number GSE120575.

### Statistics

Statistical analysis was performed using unpaired or paired *t* test for comparisons between two groups. All tests were two-sided and *P* <0.05 was considered statistically significant. Data are presented as mean ± SEM.

## Results

### Frequency, transcriptomic states, and clinical relevance of CX3CR1^+^ CD8^+^ T cells in human melanomas

**Figure 1:**
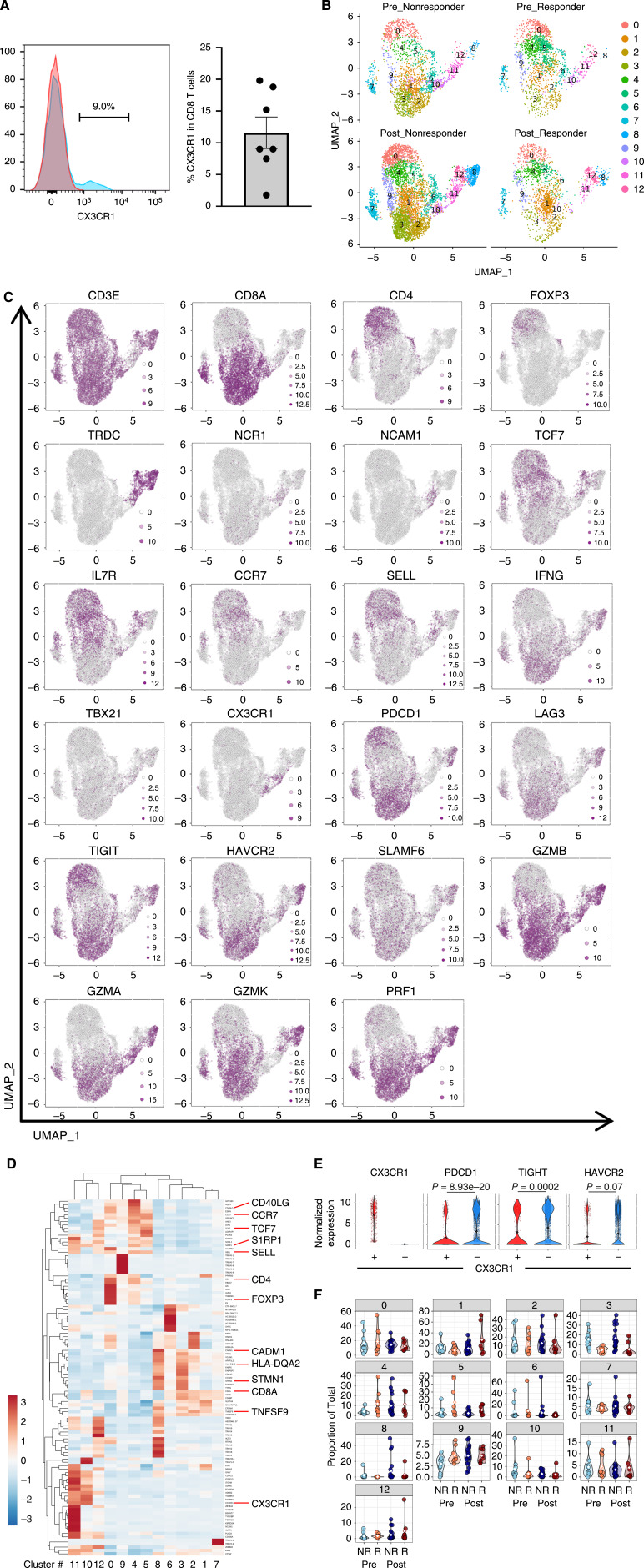
Frequency, transcriptomic phenotype, and clinical relevance of CX3CR1^+^ CD8^+^ T cells in human melanomas. **A,** Representative histogram showing CX3CR1 expression of CD8^+^ T cells in human melanomas (blue). Isotype-matched controls are shown in red. Gating strategy is shown in Supplementary Fig. S2. The right shows frequency of CX3CR1^+^ subset in CD8^+^ T cells (*n* = 7). **B–F,** Single-cell profiling of melanoma-infiltrating T and NK cells from patients treated with ICI therapy in the Sade-Feldman et al. data. Final cell-type annotations of total cells and subsequent filtering of T and NK- cells are shown in Supplementary Fig. S4A and S4B. **B,** UMAP plots of melanoma-infiltrating T and NK cells from responders and nonresponders before and after ICI therapy. **C,** Expression patterns of indicated genes in T- and NK-cell clusters in melanoma. Expression levels are color-coded: gray, not expressed; purple, expressed. **D,** Heatmap displaying the top 10 significantly enriched genes found in each cluster. **E,** Violin plots show expression of *PDCD1*, *TIGIT*, and *HAVCR2* in CX3CR1^+^ and CX3CR1^−^ populations. Differential expression is determined by Wilcoxon rank sum test. **F,** Proportions of cell clusters observed in individual melanomas from responders (R) and nonresponders (NR) at pretreatment (Pre) and post-treatment (Post).

Our study and others have reported subsets of CD8^+^ T cells expressing CX3CR1 in human melanoma tissues (15, 17). However, the frequency, transcriptomic signature, and clinical relevance of CX3CR1^+^ CD8^+^ T cells in tumors remain unclear. To this end, we evaluated surgically resected non-lymphoid primary or metastatic melanoma from seven patients using flow cytometry (Supplementary Fig. S2) and found that approximately 11% (range 1.8%–19.8%) of CD8^+^ TILs expressed CX3CR1 (Fig. 1A).

To assess the transcriptomic signatures of CX3CR1^+^ CD8^+^ T cells in the tumor microenvironment, we analyzed the scRNA-seq dataset (GSE120575), where 48 tumor samples were collected at baseline and/or during ICI therapy from 32 patients (11). Clustering analysis and subsequent cell type annotation using the human primary cell atlas (28) revealed four clusters (T and NK cells, plasmacytoid dendritic cells (pDC), B cells, and myeloid cells; Supplementary Fig. S4A–S4C).

From the initial analysis of total cells, we isolated T and NK cells to evaluate the relationship between *CX3CR1* expression and various markers in pre- and post-treatment samples from responders and non-responders (Fig. 1B–F; Supplementary Table S2). We identified 13 clusters, including two clusters expressing *CX3CR1*: cluster #10 (CD8 T cells) and cluster #11 (NK/gamma delta (γδ) Τ cells). These clusters were separated from clusters #2, 3, and 8, which expressed genes linked to cell exhaustion, such as *PDCD1, LAG3, HAVCR2, TIGIT, CD38 and ENTPD1* whereas clusters #10/11 and #2/3/8 expressed *IFNG, GZMA, GZMB and PRF1*, indicating effector T cells (Fig. 1B–D; Supplementary Table S2). Importantly, *CX3CR1* expression in human melanoma-infiltrating CD8^+^ T cells was inversely correlated with the expression of *PDCD1, TIGIT, and HAVCR2* (Fig. 1E; Supplementary Table S3), which is consistent with our previous flow cytometric analyses of mouse melanoma-specific CD8^+^ T cells and human melanoma CD8^+^ TILs (15).

Next, we sought to assess the clinical relevance of CX3CR1^+^ CD8^+^ TILs and examined whether the presence of this subset in the tumor microenvironment would correlate with the response to ICI therapy. To this end, we evaluated the frequency of each cluster in pretreatment and post-treatment samples stratified by the therapy response status (Fig. 1B and F). We found that TILs from responders exhibited an increased pretreatment frequency in clusters #4 and #5, which had enhanced expression of genes associated with memory, early activation, and cell survival, such as *IL7R*, *TCF7*, *CCR7*, *S1PR1*, *SELL*, *LTB*, and *FOXP1* as previously reported (11). Cluster #10 CD8^+^ TILs expressing *CX3CR1* pretreatment or post-treatment did not correlate with the response or resistance to ICI therapy. In contrast, a higher pretreatment and post-treatment frequency of cluster #3 CD8^+^ TILs displaying high levels of exhaustion was associated with poor response to ICI therapy.

### The differentiation of antigen-specific CX3CR1^−^ CD8^+^ T cells into CX3CR1^+^ CD8^+^ T cells *in vivo*

**Figure 2:**
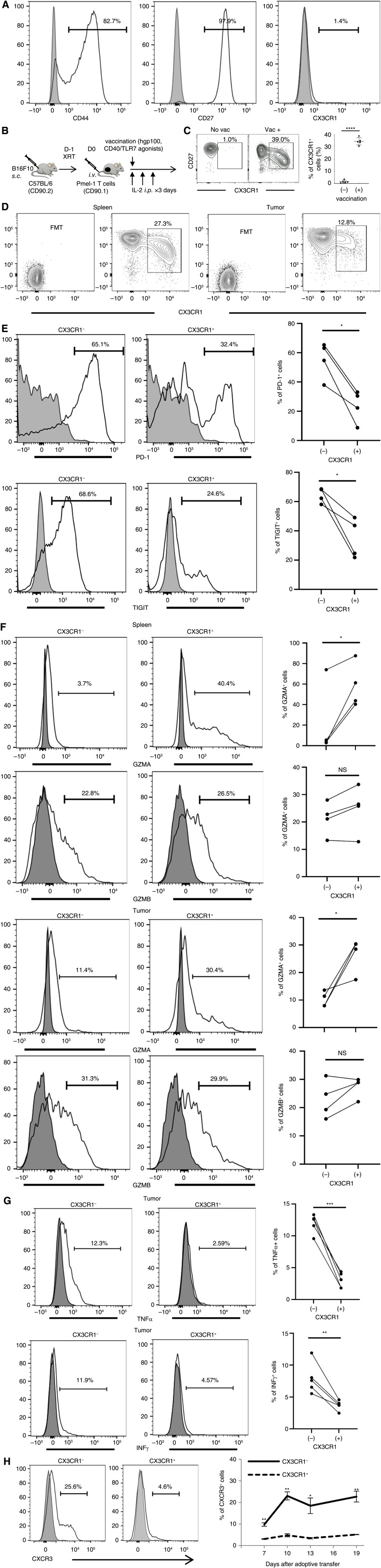
The differentiation of antigen-specific CX3CR1^−^ CD8^+^ T cells into CX3CR1^+^ CD8^+^ T cells *in vivo*. **A,** Phenotypic analysis of Pmel-1 CD8^+^ T cells after *ex vivo*-activation with hgp100 peptide, IL-7, and IL-15. **B,** Experimental protocol. XRT, external beam radiation therapy. **C,** Representative contour plots of Pmel-1 CD8^+^ T cell population in the spleen with or without vaccination. The right panels show frequency of the CX3CR1^+^ subset in CD90.1^+ ^CD8^+^ T cells (*n* = 5). (**D**) Representative contour plots of Pmel-1 CD8^+^ T cell population in the spleen and tumors 10 days after vaccination. FMT, fluorescence minus two (CD27 and CX3CR1). **E,** Representative histogram showing PD-1 and TIGIT expressions in intratumoral CX3CR1^− ^and CX3CR1^+^ CD8^+^ T cells 19 days after vaccination. Gating strategy is shown in Supplementary Fig. S3. **F,** Representative histogram showing granzyme A (GZMA) and granzyme B (GZMB) expressions in CX3CR1^− ^and CX3CR1^+^ CD8^+^ T cells in the spleen and tumors 25 days after immune vaccination. **G,** Representative histogram showing TNFα or IFNγ expression in intratumoral CX3CR1^− ^and CX3CR1^+^ CD8^+^ T cells 7 days after vaccination. **E–G,** The right panels show frequency of each T-cell subset based on CX3CR1 expression (*n* = 4–5). **H,** Representative histogram showing CXCR3 expression in intratumoral CX3CR1^− ^and CX3CR1^+^ CD8^+^ T cells 10 days after vaccination. The right panels show kinetics and frequency of the CXCR3^+ ^subset based on CX3CR1 expression. Mean (±SEM) (*n* = 4). **A** and **E–H,** Isotype-matched controls are shown in gray. **C** and **E–H,** *, *P* < 0.05; **, *P *< 0.01; ***,* P *< 0.001, and ****,* P *< 0.0001 by two-tailed unpaired (**C**) or paired (**E–H**) *t*-test. N.S., not significant.

Although CX3CR1^+^ CD8^+^ T cells are present in tumors, it remains unclear whether they directly differentiate from the CX3CR1^−^ subset within the tumor microenvironment or are trafficked from secondary lymphoid organs via the CX3CL1/CX3CR1 chemokine axis. To this end, we used Pmel-1 TCR transgenic CD8^+^ T cells (29) that can recognize gp100 expressed on B16 tumor cells lacking CX3CL1 expression (30) and evaluated the potential intratumoral differentiation of tumor-specific CD8^+^ T cells. After *ex vivo* activation with hgp100 peptide, IL7, and IL15, Pmel-1 T cells upregulated CD44 and CD27 but not CX3CR1 (Fig. 2A). However, vaccination with hgp100 and CD40/TLR7 agonists induced the differentiation of infused CX3CR1^−^ CD8^+^ T cells into CX3CR1^+^ CD8^+^ T cells *in vivo* (Fig. 2B–D; Supplementary Fig. S3A and S3B), as described previously (15).

The expression of PD-1 and TIGIT in the CX3CR1^+^ subset was substantially lower than that in the CX3CR1^−^ subset in the tumor microenvironment (Fig. 2E), in agreement with the scRNA-seq analyses of human melanoma (Fig. 1E). Next, we examined the functionality of two subsets of Pmel-1 CD8^+^ T cells. We found that GZMA expression was higher in CX3CR1^+^ CD8^+^ T cells compared to CX3CR1^−^ CD8^+^ T cells in both spleen and the tumor while expression of GZMB was equivalent (Fig. 2F). However, the expression of TNFα and IFNγ in the CX3CR1^+^ subset was substantially lower than the CX3CR1^−^ subset in the tumor (Fig. 2G). Lastly, we evaluated the expression of CXCR3 in Pmel-1 T cells where a previous study showed a nonredundant role for CXCR3 in trafficking of murine and human T cells in melanoma during adoptive cell therapy (31). CXCR3 expression remained markedly lower in the CX3CR1^+^ subset than in the CX3CR1^−^ subset of Pmel-1 T cells in the spleen (Fig. 2H), suggesting that peripheral CX3CR1^+^ Pmel-1 T cells have a decreased capacity to migrate to the tumor microenvironment via the CXCR3-CXCL9/CXCL10 axis (31).

### The differentiation of CX3CR1^−^ to CX3CR1^+^ CD8^+^ T cells could occur in the tumor without migration to secondary lymphoid organs

**Figure 3:**
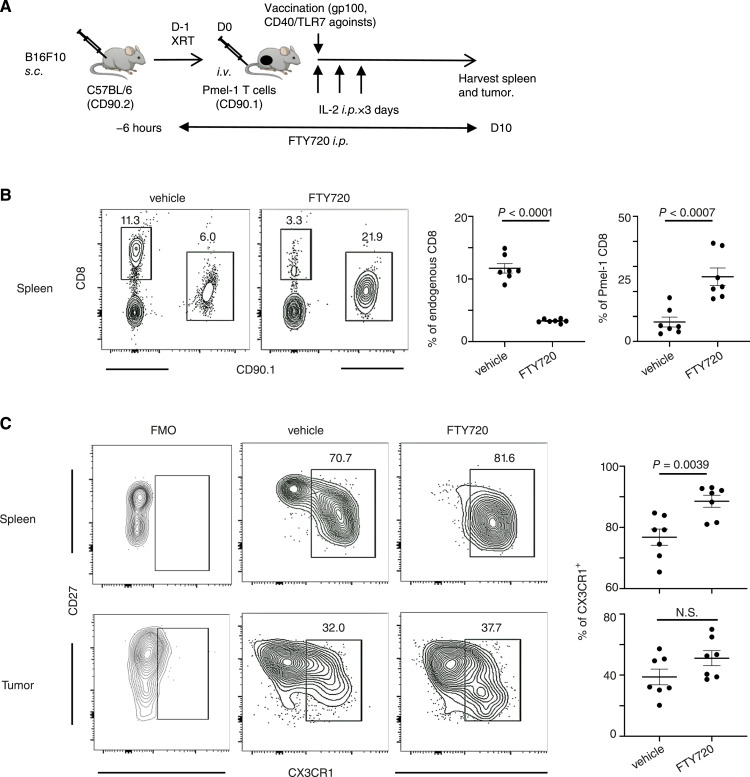
The differentiation of CX3CR1^−^ to CX3CR1^+^ CD8^+^ T cells could occur in the periphery without migration to secondary lymphoid organs. **A,** Experimental protocol. **B,** Frequency of endogenous CD90.1^−^ CD8^+^ T cells and infused Pmel-1 CD90.1^+^ CD8^+^ T cells in the spleen was analyzed after treatment with FTY720 or control vehicle. Data right show the frequency of endogenous CD90.1^−^ CD8^+^ T cells and infused Pmel-1 CD90.1^+^ CD8^+^ T cells. **C,** Representative contour plots showing CD27/CX3CR1 expression of CD8 and CD90.1-gated cells in the spleen (top) and the tumor (bottom). Numbers, percentage of the CX3CR1^+^ subset. Data right show the frequency of CX3CR1^+^ CD8^+^ T cells obtained from seven mice in each group. *P* values by two-tailed unpaired *t* test. NS, not significant. Mean (±SEM).

Next, to evaluate the role of secondary lymphoid organs in the differentiation of tumor-specific CD8^+^ T cells, we used FTY720 treatment to block sphingosine 1-phosphate receptor-1 and inhibit the migration of effector T cells from secondary lymphoid tissue (32, 33). Administration of FTY720 to B16F10-bearing mice (Fig. 3A) reduced the number of endogenous CD90.1^−^ CD8^+^ T cells and increased the number of infused CD90.1^+^ CD8^+^ Pmel-1 T cells in the spleen (Fig. 3B). As expected, FTY720 treatment increased the frequency of CX3CR1^+^ CD90.1^+^ CD8^+^ T cells in the spleen, likely because the infused CX3CR1^−^ subset differentiated upon immunization with hgp100 and CD40/TLR7 agonists (15) but could not egress from the spleen (Fig. 3C). However, CX3CR1^+^ subsets of CD90.1^+^ CD8^+^ Pmel-1 T cells were present in the tumors of FTY720-treated mice, suggesting that differentiation of CX3CR1^+^ CD8^+^ T cells occurs in the tumor and does not require the migration of CX3CR1^−^ CD8^+^ T cells to secondary lymphoid organs.

### Antigen-specific CX3CR1^−^ CD8^+^ T cells differentiate to CX3CR1^+^ subsets within the tumor

**Figure 4:**
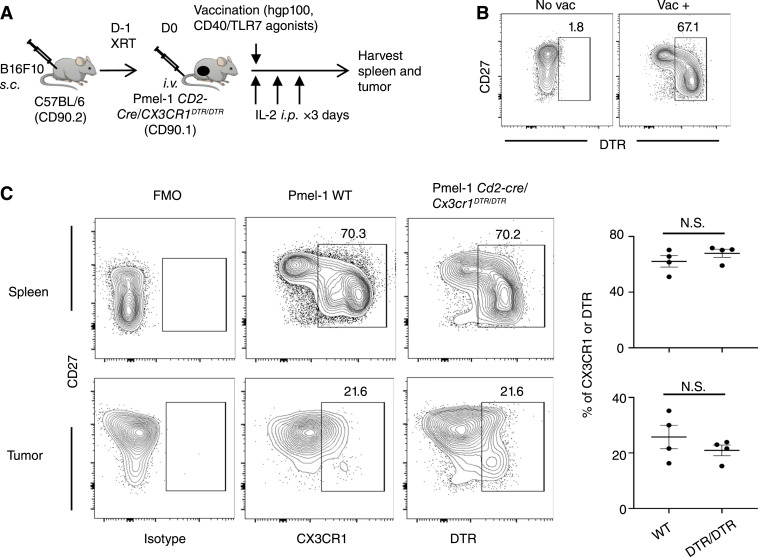
Antigen-specific CD8^+^ T cells CX3CR1^−^ CD8^+^ T cells differentiate to CX3CR1^+^ subsets within the tumor. **A,** Experimental protocol. B16F10-bearing mice were either injected with *ex vivo*-activated Pmel-1 CD8^+^ T cells (WT) or Pmel-1 *Cd2-cre/Cx3cr1*^*DTR/DTR*^ CD8^+^ T cells. The scheme of generation of Pmel-1 *Cd2-cre/Cx3cr1*^*DTR/DTR*^ mice is shown in Supplementary Fig. S1. **B,** Representative contour plots showing CD27/DTR expression of CD8 and CD90.1-gated *Cd2-cre/Cx3cr1*^*DTR/DTR*^ CD8^+^ T cells in the spleen with or without vaccination with hgp100 and CD40/TLR7 agonists *in vivo*. **C,** Representative contour plots of CD8 and CD90.1-gated cells in the spleen (top) and the tumor (bottom). Numbers, percentage of the CX3CR1^+^ subset. Data panels right show frequency of CX3CR1^+^ CD8^+^ T cells [wild type (WT)] or DTR-expressing Pmel-1 *Cd2-cre/Cx3cr1*^*DTR/DTR*^ CD8^+^ T cells (DTR/DTR) obtained from four mice in each group. NS, not significant by two-tailed unpaired *t* test.

Given the decreased expression of CXCR3 in peripheral CX3CR1^+^ subsets (Fig. 2H) and the presence of the CX3CR1^+^ subset in the tumor despite FTY720 administration (Fig. 3C), we hypothesized that CX3CR1^+^ subsets could directly differentiate from the CX3CR1^−^ subset *in situ* rather than trafficking to the tumor mediated via the CX3CR1/CX3CL1 pathway. To test this hypothesis, we generated Pmel-1 *Cd2-cre/Cx3cr1*^*DTR/DTR*^ mice, in which the CX3CR1 gene was mutated and replaced with DTR in Pmel-1 CD8^+^ T cells. Homozygous Pmel-1 *Cd2-cre/Cx3cr1*^*DTR/DTR*^ mice lack CX3CR1 but express DTR in ‘‘wannabe’’ CX3CR1^+^ Pmel-1 CD8^+^ T cells. Although Pmel-1 *Cd2-cre/Cx3cr1*^*DTR/DTR*^ CD8^+^ T cells do not have the function of CX3CR1 as a chemokine receptor for trafficking to the tumor via the CX3CR1/CX3CL1 axis, terminally differentiated CD8^+^ T cells with an active CX3CR1 promoter can still be identified using an anti-DTR antibody if residing in the tumor. Thirteen days after wild-type and *Cd2-cre/Cx3cr1*^DTR/DTR^ Pmel-1 CD8^+^ T cells were adoptively transferred, we evaluated their CX3CR1 and DTR expression in the spleen and tumors of recipient B16F10-bearing C57BL/6 mice (Fig. 4A). Upon vaccination with hgp100 and CD40/TLR7 agonists, adoptively transferred *Cd2-cre/Cx3cr1*^DTR/DTR^ Pmel-1 CD8^+^ T cells expressed DTR *in vivo* (Fig. 4B). In both the spleen and tumor, the frequency of CX3CR1^+^ in wild-type Pmel-1 CD8^+^ T cells was comparable to that of DTR-expressing cells in Pmel-1 *Cd2-cre/Cx3cr1*^*DTR/DTR*^ CD8^+^ T cells, indicating that the presence of differentiated antigen-specific CD8^+^ T cells within the tumor is independent of the presence of CX3CR1 (Fig. 4C). Collectively, these data support the notion that CX3CR1^+^ CD8^+^ T cells differentiate directly from intratumoral CX3CR1^−^ CD8^+^ T cells and that T-cell differentiation occurs within the tumor microenvironment.

## Discussion

The developmental pathway of intratumoral T cells has been under intensive investigation since the discovery of immune checkpoints, such as PD-1, expressed on T cells. Although recent advances in single-cell profiling have allowed the identification of various T-cell states, the differentiation trajectory of intratumoral CD8^+^ T cells is difficult to determine. In this study, using a preclinical model of melanoma that lacks CX3CL1 expression (30) and tumor-specific CD8^+^ T cells in which the *CX3CR1* gene was replaced with a functionally unrelated gene, we have demonstrated the intratumoral generation of antigen-specific CX3CR1^+^ CD8^+^ T cells. Moreover, using surgically resected specimens and a publicly available database of human melanomas, we have provided insights into the frequency, transcriptomic signatures, and clinical relevance of CX3CR1^+^ CD8^+^ T cells in human melanomas.

Recent advances in single-cell methods and technology have revealed that a vast heterogeneity of intratumoral T-cell states exists in cancers (34). Upon analysis of a publicly available single-cell dataset, we confirmed the presence of a cluster expressing *CX3CR1*, which was distinct from CD8 T-cell clusters expressing markers of exhaustion. These findings align with the model in which exhausted T cells are not terminally differentiated states derived from late-stage effector cells but are rather composed of populations that develop progressively from a deviated pattern of memory T-cell differentiation during continuous antigen stimulation (35–37). Furthermore, our finding of an inverse correlation between *CX3CR1* and *PDCD1* expression in human melanoma-infiltrating CD8^+^ T cells confirmed our previous observation from flow cytometric analyses (15) and aligned with the divergent fate commitment to short-lived differentiated effector T cells and exhausted T cells (38).

Although our results indicate the differentiation of tumor-specific CD8^+^ T cells in the tumor, the antitumor reactivity of CX3CR1^+^ CD8^+^ T cells remains unclear. Previous studies using CX3CR1^−/−^ mice or CX3CR1 antagonists have shown a potential role of CX3CR1-expressing cells in antitumor immunity (17, 30, 39–41). However, our recent studies using selective depletion of CX3CR1^+^ T cells did not demonstrate the contribution of this subset to effective adoptive cell therapy and vaccine-based therapy (15, 21). In line with this, we found markedly decreased TNFα and IFNγ expressions of CX3CR1^+^ CD8^+^ T cells (Fig. 2F). This scenario aligns with a growing body of evidence indicating that less-differentiated CD8^+^ T cells retain polyfunctionality, produce effector cytokines, persist longer, and provide greater control of viral infection (42–44) and established tumors (6, 7) than fully differentiated effector T cells *in vivo*. In the context of immune checkpoint blockade therapy, an increased frequency of less-differentiated memory T cells with higher expression of TCF-1 (encoded by *Tcf7*) in the tumor microenvironment was correlated with response to the treatment and better survival in preclinical models and human melanomas (11, 45, 46). Our results from scRNA-seq analysis in the present study was in agreement with these previous findings and further demonstrated that expression of *CX3CR1* in CD8^+^ TILs pretreatment or post-treatment did not correlate with the response or resistance to ICI therapy (Fig. 1F). Therefore, CX3CR1 may be a marker of short-lived differentiated effector T cells with limited contribution to the antitumor immunity.

In the present study, FTY720 treatment eliminated CX3CR1^−^ CD8^+^ cells in the periphery but not in the tumor (Fig. 3C). These results suggest that CX3CR1^−^ CD8^+^ cells are directly trafficked to the tumor microenvironment, and differentiate into the CX3CR1^+^ subset. However, the mechanisms underlying the intratumoral differentiation of CD8^+^ T cells remain unclear. This might occur upon encountering the antigen expressed on tumors and/or hgp100-pulsed myeloid cells, while this might be mediated by the tertiary lymphoid structure in B16 melanoma tumors (47). Further studies with spatial analysis of the tumor microenvironment are warranted to elucidate antigen presentation in the tumor microenvironment that facilitates the differentiation of CD8^+^ T cells.

The present study had several limitations. We used only one mouse B16F10 melanoma tumor model. We focused on the differentiation capacity and did not evaluate other functions of Pmel-1 *Cd2-cre/Cx3cr1*^*DTR/DTR*^ CD8^+^ T cells compared to wild-type Pmel-1 CD8^+^ T cells. The differentiation of CD8^+^ T cells was assessed only in the spleen and tumors in the setting of adoptive cell therapy. Similarly, the components of CD8^+^ T-cell states may vary with different types of immunotherapies (vaccine-based therapy, etc.) and the model used (immunogenic vs. non-immunogenic tumors). Notably, our study does not negate the ability of CX3CR1^+^ CD8^+^ T cells to traffic to the tumor microenvironment if tumors secrete CX3CL1. Indeed, a previous study showed that enhanced expression of CX3CL1 in the tumor improves control of the established tumor in multiple preclinical models, including B16 melanoma, by NK-cell– and T-cell–dependent mechanisms (30, 48, 49). An increased frequency of circulating CX3CR1^+^ CD8^+^ T cells has been found in effective immunotherapies such as adoptive T cell therapy, vaccine-based therapy, and ICI therapy in preclinical models and patients (15–21). However, it remains elusive whether the change in the frequency of intratumoral CX3CR1^+^ CD8^+^ T cells is a predictor of response to immunotherapy. Although our analysis suggests that the increased frequency of this subset might not correlate with response to immunotherapy, additional cohorts are needed to validate this finding.

In conclusion, our findings demonstrate the transcriptomic states of *CX3CR1*^+^ CD8^+^ T cells and intratumoral generation of antigen-specific CX3CR1^+^ CD8^+^ T cells. Our study provides new insights into the clinical and immunological relevance of CX3CR1 expression in the tumor microenvironment of melanoma.

## Supplementary Material

Supplementary Figure 1 The scheme of the generation of Pmel-1 Cd2-cre/Cx3cr1DTR/DTR mice.

Supplementary Figure 2 Gating strategy to analyze CX3CR1 expression on CD8+ T cells in human melanoma TILs.

Supplementary Figure 3 Gating strategy to analyze the adoptively transferred Pmel-1 T cells with CD90.1 expression.

Supplementary Figure 4 Single-cell profiling of melanoma-infiltrating cells from patients treated with immune checkpoint inhibitor therapy in the Sade-Feldman et al. data

Supplementary Table 1 Supplementary Table 1

Supplementary Table 2 Supplementary Table 2

Supplementary Table 3 Supplementary Table 3
