# Supplementary material for: Generation, Transcriptomic States, and Clinical Relevance of CX3CR1+ CD8 T Cells in Melanoma
Source: Cancer Res Commun. 2024 Jul 24;4(7):1802–14. doi: 10.1158/2767-9764.CRC-24-0199 (PMC11267618; doi:10.1158/2767-9764.CRC-24-0199)
Supplement: Supplementary Figure 1 — The scheme of the generation of Pmel-1 Cd2-cre/Cx3cr1DTR/DTR mice. [file crc-24-0199_supplementary_figure_1_suppsf1.pdf]

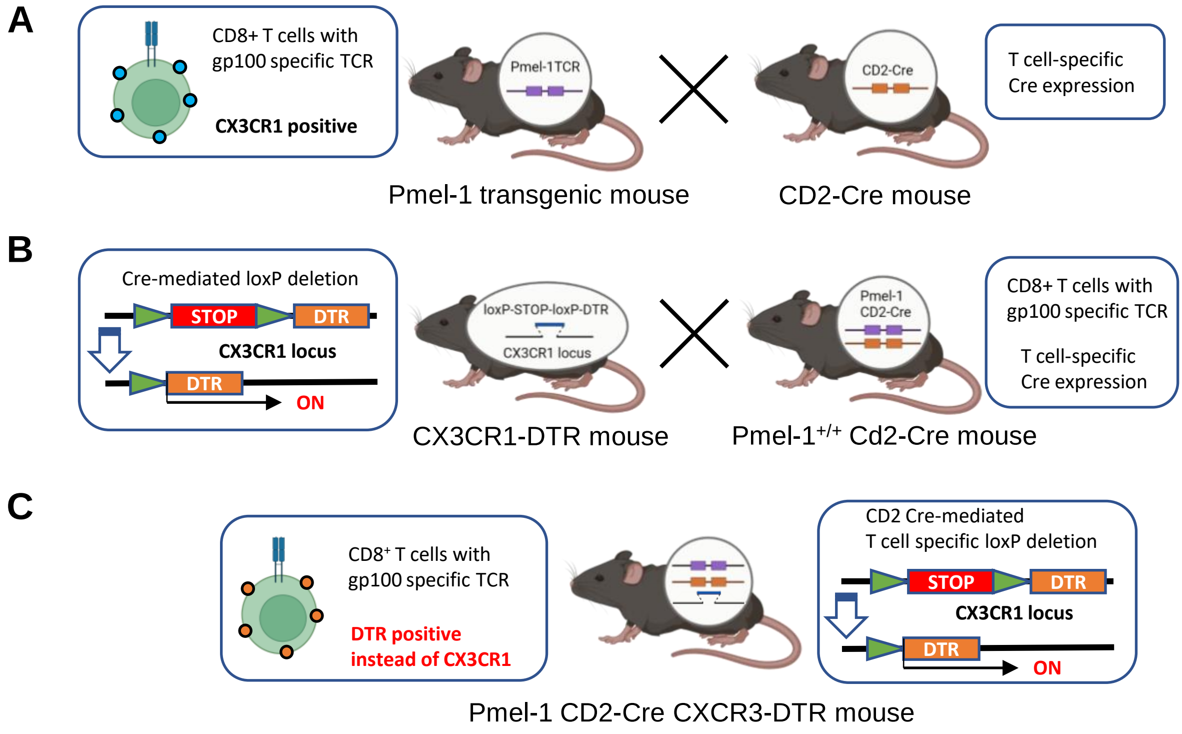

Supplementary Fig. 1. The scheme of the generation of Pmel-1 *Cd2-cre/Cx3cr1*$^{\mathit{DTR/DTR}}$ mice. Related to Fig. 4.

(**A**) Generation of Pmel-1$^{+/+}$ *Cd2-cre* mice in which Pmel-1 CD8^{+} T cells expressed Cre. Pmel-1 mice were crossed with CD2-Cre mice. Next, Pmel-1$^{+/-}$ *Cd2-cre* mice were backcrossed with Pmel-1 mice to generate Pmel-1$^{+/+}$ *Cd2-cre* mice. (**B**) Generation of Pmel-1 *Cd2-cre/Cx3cr1*$^{+/\mathrm{DTR}}$ mice by crossing Pmel-1$^{+/+}$ *Cd2-cre* mice with CX3CR1-DTR mice, in which the iDTR system was knocked in at the CX3CR1 gene locus. The generated mice showed Pmel-1 CD8^{+} T cells with a heterozygous deletion of CX3CR1. (**C**) We crossed Pmel-1 *Cd2-cre/Cx3cr1*$^{+/\mathrm{DTR}}$ mice with Pmel-1 *Cd2-cre/Cx3cr1*$^{+/\mathrm{DTR}}$ mice to generate *Cd2-cre/Cx3cr1*$^{\mathrm{DTR/DTR}}$ mice. The mouse has "wannabe" CX3CR1^{+} Pmel-1 CD8^{+} T cells with DTR expression instead of CX3CR1.
