# Supplementary material for: Generation, Transcriptomic States, and Clinical Relevance of CX3CR1+ CD8 T Cells in Melanoma
Source: Cancer Res Commun. 2024 Jul 24;4(7):1802–14. doi: 10.1158/2767-9764.CRC-24-0199 (PMC11267618; doi:10.1158/2767-9764.CRC-24-0199)
Supplement: Supplementary Figure 2 — Gating strategy to analyze CX3CR1 expression on CD8+ T cells in human melanoma TILs. [file crc-24-0199_supplementary_figure_2_suppsf2.pdf]

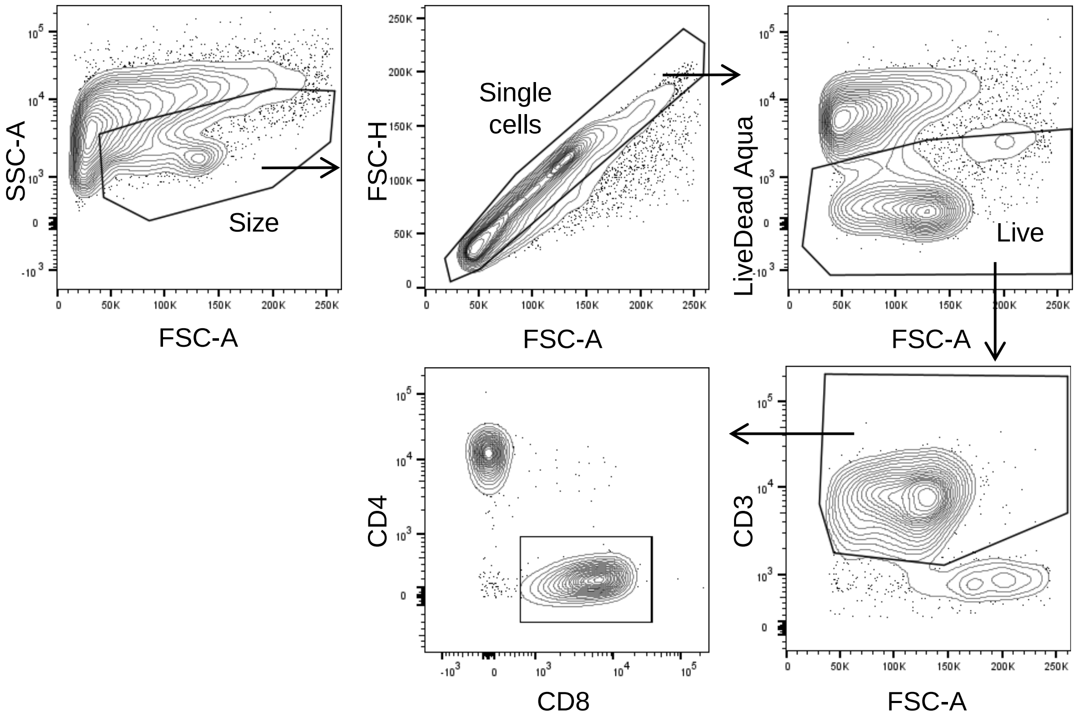

Supplementary Fig. 2. Gating strategy to analyze CX3CR1 expression on CD8$^+$ T cells in human melanoma TILs. Related to Fig. 1A.

Panels and arrows indicate the gating strategy used to analyze human melanoma-infiltrating CD8$^+$ T cells. CD3 and CD8 double-positive cells in the tumor were analyzed after gating for lymphocyte size, excluding doublets and dead cells. FSC, forward scatter; SSC, side scatter.
