# Supplementary material for: Generation, Transcriptomic States, and Clinical Relevance of CX3CR1+ CD8 T Cells in Melanoma
Source: Cancer Res Commun. 2024 Jul 24;4(7):1802–14. doi: 10.1158/2767-9764.CRC-24-0199 (PMC11267618; doi:10.1158/2767-9764.CRC-24-0199)
Supplement: Supplementary Figure 3 — Gating strategy to analyze the adoptively transferred Pmel-1 T cells with CD90.1 expression. [file crc-24-0199_supplementary_figure_3_suppsf3.pdf]

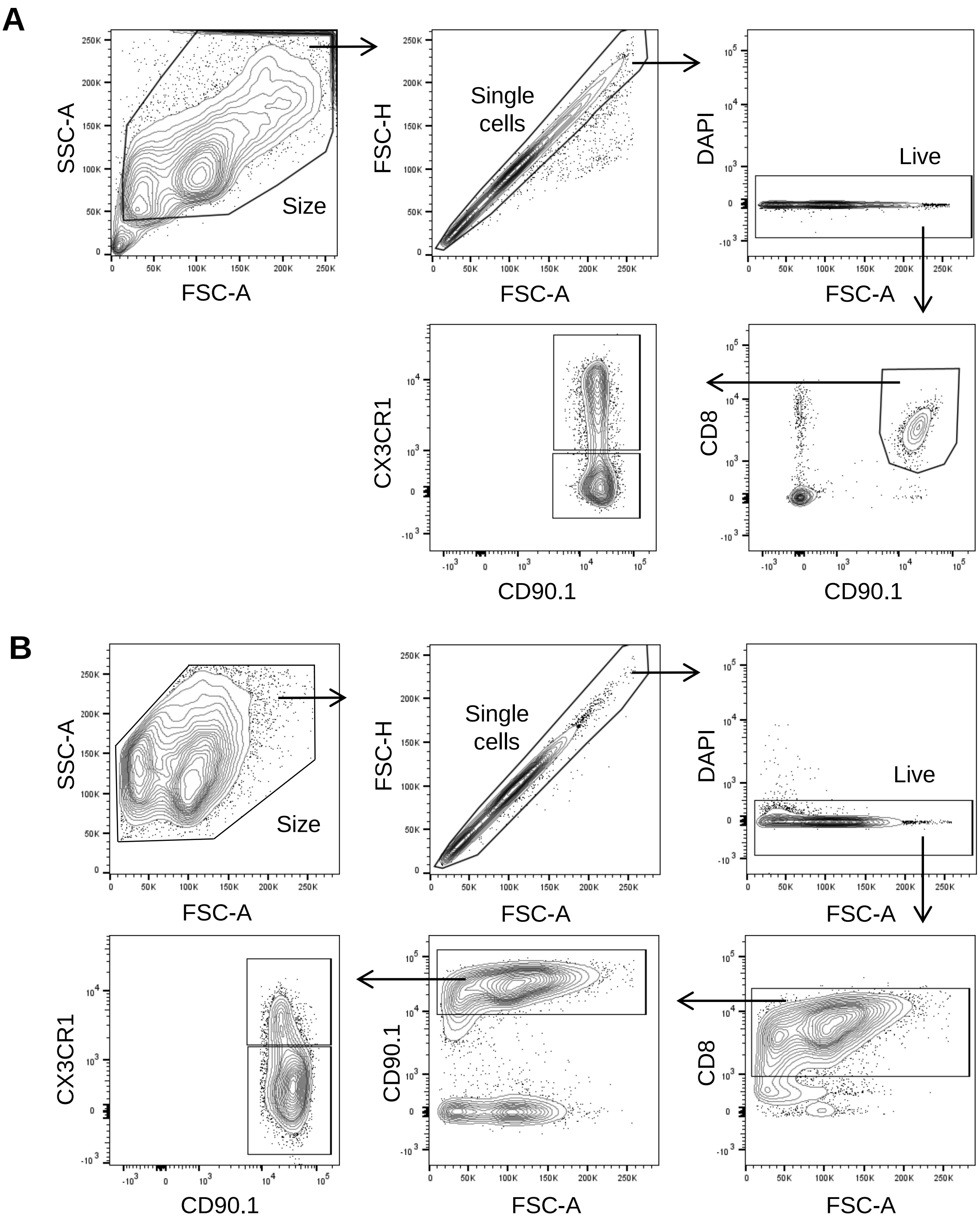

Supplementary Fig. 3. Gating strategy to analyze the adoptively transferred Pmel-1 T cells with CD90.1 expression. Related to Fig. 2.

(**A, B**) Panels and arrows indicate the gating strategy used to analyze Pmel-1 T cells in the spleen (A) and tumors (B). $CD3^{+}$ $CD8^{+}$ $CD90.1^{+}$ T cells were gated as adoptively transferred Pmel-1 T cells, after excluding doublets and dead cells. $CX3CR1^{+}$ and $CX3CR1^{-}$ cells were analyzed.
