# Supplementary material for: Generation, Transcriptomic States, and Clinical Relevance of CX3CR1+ CD8 T Cells in Melanoma
Source: Cancer Res Commun. 2024 Jul 24;4(7):1802–14. doi: 10.1158/2767-9764.CRC-24-0199 (PMC11267618; doi:10.1158/2767-9764.CRC-24-0199)
Supplement: Supplementary Figure 4 — Single-cell profiling of melanoma-infiltrating cells from patients treated with immune checkpoint inhibitor therapy in the Sade-Feldman et al. data [file crc-24-0199_supplementary_figure_4_suppsf4.pdf]

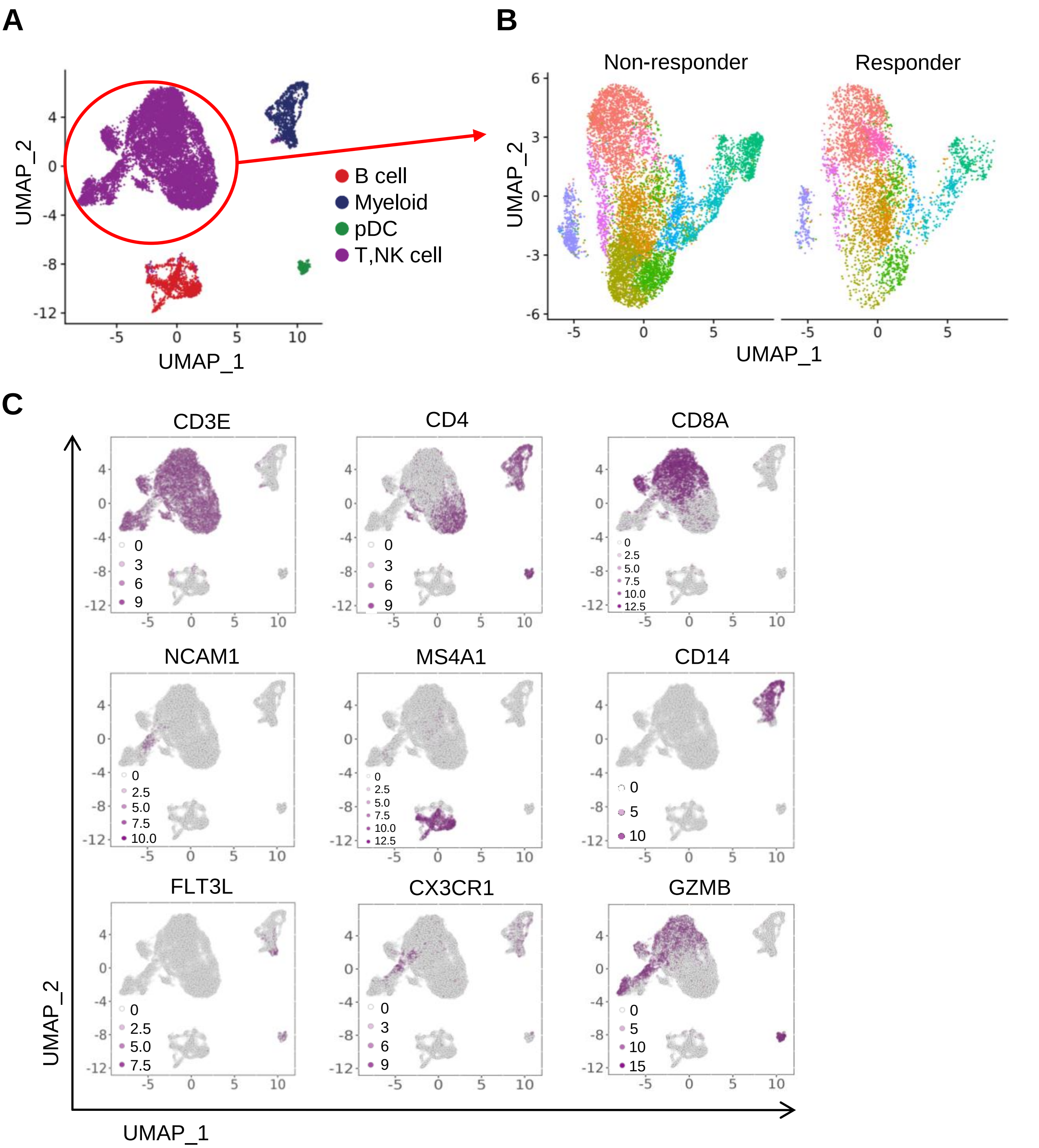

Supplementary Fig. 4. Single-cell profiling of melanoma-infiltrating cells from patients treated with immune checkpoint inhibitor therapy in the Sade-Feldman et al. data. Related to Fig. 1B-F.

(**A**) UMAP plots depicting major cell linage annotations of total melanoma-infiltrating immune cells generated by Sade-Feldman et al. (**B**) UMAP plots depicting unsupervised clustering of T and NK cells identified in Supplementary Figure 2A in non-responder and responder patients. (**C**) Expression patterns of indicated genes in UMAP space. Expression levels are color-coded: gray, not expressed; purple, expressed.
