# Supplementary material for: Generation, Transcriptomic States, and Clinical Relevance of CX3CR1+ CD8 T Cells in Melanoma
Source: Cancer Res Commun. 2024 Jul 24;4(7):1802–14. doi: 10.1158/2767-9764.CRC-24-0199 (PMC11267618; doi:10.1158/2767-9764.CRC-24-0199)
Supplement: Supplementary Table 1 [file crc-24-0199_supplementary_table_1_suppst1.pdf]

Supplementary Table 1. Antibodies for flow cytometric analysis.

	ANTIBODY	CLONE	VENDER
HUMAN	CD3	UCH1-1	BioLegend
	CD4	SK3	BD Biosciences
	CD8	RPA-T8	BD Biosciences
	CX3CR1	2A9-1	BD Biosciences
MOUSE	CD8	53-6.7	BioLegend
	CD90.1	OX-7	BioLegend
	CX3CR1	SA011F11	BioLegend
	CD27	LG.7F9	Thermo Fisher
	CD44	IM7	BioLegend
	CXCR3	CXCR3-173	BioLegend
	PD-1	29F.1A12	BioLegend
	TIGIT	1G9	BioLegend
	Granzyme A	GzA-3G8.5	Thermo Fisher
	Granzyme B	16G6	Thermo Fisher
	TNFα	MP6-XT22	BioLegend
	IFNγ	XMG1.2α	Thermo Fisher
